# Design-development of an at-home modular brain–computer interface (BCI) platform in a case study of cervical spinal cord injury

**DOI:** 10.1186/s12984-022-01026-2

**Published:** 2022-06-03

**Authors:** Kevin C. Davis, Benyamin Meschede-Krasa, Iahn Cajigas, Noeline W. Prins, Charles Alver, Sebastian Gallo, Shovan Bhatia, John H. Abel, Jasim A. Naeem, Letitia Fisher, Fouzia Raza, Wesley R. Rifai, Matthew Morrison, Michael E. Ivan, Emery N. Brown, Jonathan R. Jagid, Abhishek Prasad

**Affiliations:** 1 Department of Biomedical Engineering, University of Miami, 1251 Memorial Dr, MEA 204, Coral Gables, Miami, FL 33146 USA; 2 Department of Brain and Cognitive Science, Massachusetts Institute of Technology, Cambridge, MA 02139 USA; 3 Department of Anesthesia, Critical Care and Pain Medicine, Massachusetts General Hospital, Boston, MA 02114 USA; 4 Picower Institute for Learning and Memory, Massachusetts Institute of Technology, Cambridge, MA 02139 USA; 5 Department of Neurological Surgery, University of Miami, 1095 NW 14th Terrace, Miami, FL 33136 USA; 6 Department of Electrical and Information Engineering, University of Ruhuna, Matara, Sri Lanka; 7 Department of Biomedical Engineering, Georgia Institute of Technology, Atlanta, GA 30332 USA; 8 Division of Sleep Medicine, Harvard Medical School, Boston, MA 02115 USA; 9 Miami Project to Cure Paralysis, University of Miami, Miami, FL 33136 USA; 10 Harvard John A. Paulson School of Engineering and Applied Sciences, Harvard University, Cambridge, MA 02138 USA

**Keywords:** Neuroscience, Rehabilitation, Signal processing systems

## Abstract

**Objective:**

The objective of this study was to develop a portable and modular brain–computer interface (BCI) software platform independent of input and output devices. We implemented this platform in a case study of a subject with cervical spinal cord injury (C5 ASIA A).

**Background:**

BCIs can restore independence for individuals with paralysis by using brain signals to control prosthetics or trigger functional electrical stimulation. Though several studies have successfully implemented this technology in the laboratory and the home, portability, device configuration, and caregiver setup remain challenges that limit deployment to the home environment. Portability is essential for transitioning BCI from the laboratory to the home.

**Methods:**

The BCI platform implementation consisted of an Activa PC + S generator with two subdural four-contact electrodes implanted over the dominant left hand-arm region of the sensorimotor cortex, a minicomputer fixed to the back of the subject’s wheelchair, a custom mobile phone application, and a mechanical glove as the end effector. To quantify the performance for this at-home implementation of the BCI, we quantified system setup time at home, chronic (14-month) decoding accuracy, hardware and software profiling, and Bluetooth communication latency between the App and the minicomputer. We created a dataset of motor-imagery labeled signals to train a binary motor imagery classifier on a remote computer for online, at-home use.

**Results:**

Average bluetooth data transmission delay between the minicomputer and mobile App was 23 ± 0.014 ms. The average setup time for the subject’s caregiver was 5.6 ± 0.83 min. The average times to acquire and decode neural signals and to send those decoded signals to the end-effector were respectively 404.1 ms and 1.02 ms. The 14-month median accuracy of the trained motor imagery classifier was 87.5 ± 4.71% without retraining.

**Conclusions:**

The study presents the feasibility of an at-home BCI system that subjects can seamlessly operate using a friendly mobile user interface, which does not require daily calibration nor the presence of a technical person for at-home setup. The study also describes the portability of the BCI system and the ability to plug-and-play multiple end effectors, providing the end-user the flexibility to choose the end effector to accomplish specific motor tasks for daily needs.

*Trial registration* ClinicalTrials.gov: NCT02564419. First posted on 9/30/2015

## Introduction

Paralysis is a devastating condition that affects approximately 5.4 million people in the US alone [1]. Among the various causes that result in paralysis, stroke is the most common cause followed by spinal cord injury (SCI) and multiple sclerosis [2]. For SCI, incidence is highest in the US and the prevalence of cervical SCI is rising [3]. Paralysis imposes a significant economic and social burden on the individual, their families, caregivers, and public health, as the costs for individuals with high tetraplegia can exceed $1 M in the 1st year after injury [4]. For 40% of stroke subjects, and most SCI subjects, functional deficits are typically permanent, and no treatment yet exists. Thus, the need to address functional improvements and restoration of movement and independence in these subjects remains a critical challenge.

Despite a lack of available treatment, recent advances in intracortical brain–computer interfaces (BCI) have shown promising results in restoring functional reaching and grasping in individuals with paralysis [5–8]. BCIs create an external link between the brain and body by means other than the body’s nervous system [9]. While BCIs may target a variety of consumers, this technology yields promising outcomes for subjects with sensorimotor deficits, in which BCIs can circumvent lost function [10].

Research efforts investigating invasive BCIs have highlighted their potential to restore function lost due to neurological disorder and injury. Invasive neural recordings have enabled human subjects to control virtual cursors [11–16], computers [17, 18], spellers [19], virtual [20–22] and robotic prosthetics [6–8, 15, 23], exoskeletons [24], and their own paralyzed limbs via functional electrical stimulation (FES) [5, 25, 26]. These achievements highlight many remarkable improvements in BCIs, such as the ability of neural-control devices in multiple degrees of freedom [8, 15, 23, 24]. These demonstrations generally require subjects to be tethered to the neural data acquisition hardware to allow for high-resolution data streaming [6–8, 12–15, 17, 20–22, 25, 26]. However, the constant need to tether the subject to the data acquisition hardware limits the subject’s ability to use the BCI in their home and community, subsequently providing no real way for the subjects to control or configure how they interact with the device. Therefore, progress in the field to enable BCIs to function outside of a research environment remains a critical milestone [27, 28].

The development of portable BCIs for out-of-lab use, or BCI systems that are not tethered to large stationary research equipment and can be easily transported anywhere, remains a significant challenge for intracortical BCIs as they must consider the constraints that current wireless technologies impose on high-data-rate wireless telemetry, ultra-low-power electronics, and space that the physical components occupy, both inside and on the surface of the body [29, 30].

Research in animal studies continue to help solve these problems by developing wireless intracortical interfaces [31] and modifying device parameters that decrease power consumption without significant impacts on decoder quality [32]. Recently, Simeral and colleagues demonstrated an untethered approach of an intracortical BCI for controlling a computer cursor—relying on a short-range radio frequency (RF) receiver tower placed in the same room as the subject to capture high-bandwidth data transmission from a wireless RF head-mount [33].

Notably, many studies that utilize electroencephalography (EEG) have accomplished home use in a variety of applications [34–38]; however, EEG-based BCIs lack the spatial resolution captured by more invasive techniques [11]. Moreover, the use of non-invasive technologies, such as EEG, for BCI control signals can complicate set up procedures (e.g., appropriate electrode placement) limiting the subject’s independence and the ease of BCI use at home.

Though invasive intracortical microelectrode arrays have excellent signal quality and have demonstrated many of the remarkable strengths of BCI capablities, these arrays suffer from diminished signal quality over time, typically as a result of the inflammation triggered by the foreign body response to the electrode shanks puncturing the blood–brain barrier [39–42]. These devices are often percutaneous implants, where a portion of the implant is fully exposed increasing the risk of infection [43].

Similar to intracortical micro arrays are the use of invasive electrodes on the surface of the brain via electrocorticography (ECoG). This method has enabled long-term and stable signal acquisition [44] with higher spatial resolution and signal-to-noise ratio [45]. Such applications have successfully controlled cursors [11, 46], computers [18], spellers [47], and recently exoskeletons [24] with multiple degrees of freedom. Chronic, fully implanted implementations have enabled the translation of research out of the lab and into the home [19, 47]. While fully implantable BCIs require the need for invasive brain surgery, they have the potential to move applicable BCI technology into the home setting, by minimizing set up time for caregivers and minimizing percutaneous infections since no part of the implanted device is exposed, as is the case in intracortical microelectrode arrays.

Potential users of BCI and their caregivers indicated that portability, simple system configurations, and minimized setup times are important components of BCI system implementations [48]. Addressing these design considerations will better facilitate transitions into the home. To accomplish this goal of designing at-home BCI systems, we used a minicomputer mounted on the subject’s wheelchair that wirelessly recorded ECoG data from the implanted Activa PC + S device and performed neural decoding. Our design included a modular approach, which enabled the subject to select from a variety of output devices and allowed for add-on peripheral devices in the future by utilizing a nearly plug-and-play ready system.

## Methods

### Overview of system design

All study procedures were approved by the University of Miami Institutional Review Board (IRB 20190536) and the U.S. FDA (ClinicalTrials.gov: NCT02564419).

#### Subject

The at-home BCI system was implemented in a 22-year-old subject with chronic cervical quadriplegia (C5 ASIA A) to restore hand grasp. The subject was part of a case study in which one subject enrolled with the purpose of fully implanting a neural sensing device for investigating brain–computer interfaces in the laboratory. This subject was injured about 6 years prior in a motor vehicle accident and had volitional control over their bicep muscles, but not triceps, or other distal muscle groups.

#### System implementation

The main components of the BCI consisted of a minicomputer mounted to the subject’s wheelchair, the subject’s smartphone, a neural signal acquisition device, and an end effector. The smartphone served as the user interface for the system, while the computer orchestrated the data acquisition, processing, and transmission between a signal acquisition device and an end effector.

#### Input device—neural data acquisition

**Fig. 1:**
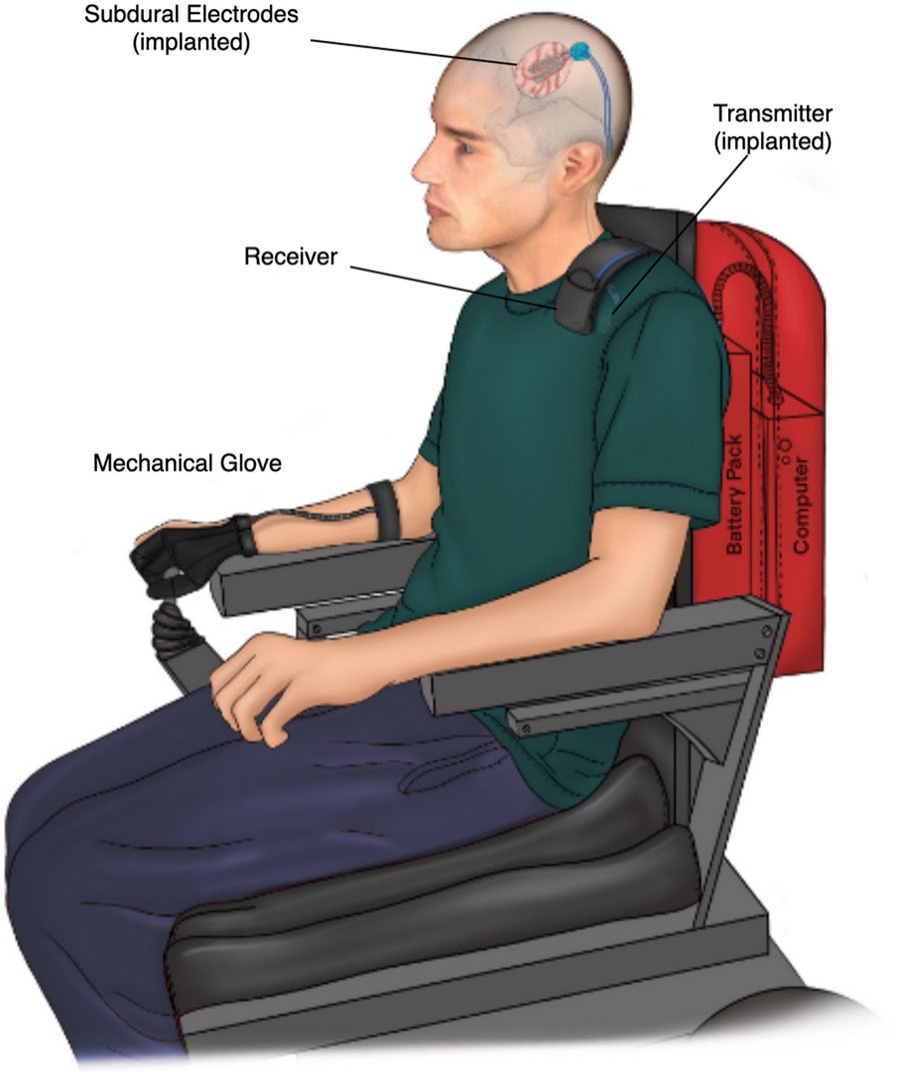
System overview. Electrocotricography (ECoG) signals are recorded using two four-contact subdural strips placed on the surface of the sensorimotor cortex. ECoG signals were transmitted by a subcutaneous implant to an external receiver which delivers it to the minicomputer for processing. The decoder classified the signal as a motor imagery command that is then sent over Bluetooth to actuate the mechanical glove

The hardware used to collect the neural data for this BCI platform [49], consisted of three parts, two internal components and one external: (1) two four-contact electrode leads (Resume II leads, Medtronic) implanted intracranially over the subdural surface of the sensorimotor hand region of the brain, (2) an Activa PC + S (Medtronic, USA) generator implanted inferior to the left clavicle for recording and transmitting the signal sensed by the contact electrodes, and (3) an external Nexus-D telemeter receiver (Medtronic, USA) which collected the transmitted signal when it’s antenna was placed in proximity to the implanted Activa PC + S generator (Fig. 1). The eight implanted electrodes were configured in a bipolar configuration resulting in four channels of ECoG data. In this configuration, the Activa PC + S allows for only two time series and two power sampling channels. Channels 1 and 3 provided real-time ECoG output sampled at 200 Hz, and channels 2 and 4 provided average power output between 4–36 Hz sampled at 5 Hz. Theses frequency settings were selected based on initial laboratory measurements for detecting event-related desynchronization [49]. Data from these channels were transmitted in packets gathered by the Nexus telemeter and delivered to the computer for further processing via a USB serial connection. The device, and its ability to detect movement intention, was rigorously tested in the laboratory setting after the subject was implanted with the device for several months prior to home deployment [49].

#### Output device—end effector

Hand grasp was actuated using a mechanical glove (Neomano, Neofect, South Korea). All input devices (neural data acquisition) and output devices (end effectors) communicated with the computer over serial port communication, while custom mobile phone application (App) and minicomputer communicated over Bluetooth Low Energy (BLE) protocol.

### Experimental setup

#### Data collection

We used the BCI platform at home to collect data to train a continuous motor imagery classifier. The subject used the mobile application to initiate data collection sessions. To gather ECoG data associated with movement intent, textual prompts were sent to the subject’s phone displaying “MOVE” or “REST” and the subject was instructed to think about opening and closing their hand rapidly during the MOVE state (Fig. 3d). The prompt alternated back and forth randomly at intervals between 6–10 s (corresponding to 15–25 packets, or 1200–2000 samples of time-series data for channels 1 and 3 collected by the PC + S). This alternating process lasted for 5 min to gather a total of 750 packets (or 60,000 samples of data per time channel) per session of data collection. 33 of these 5-min trials were used to train the decoder (summing to 165 min of training data) after which 17 open-loop trials and 12 close-loop trials were used to validate and test the decoder. Close-loop trials were trials where decoded values were actively controlling the prosthetic glove during data collection, while in open-loop trials, the prosthetic glove was not triggered. It should be noted that this move-rest protocol for training the decoder is not specific to the BCI platform but rather associated with the implementation and input device specification.

### Signal processing

**Fig. 2:**
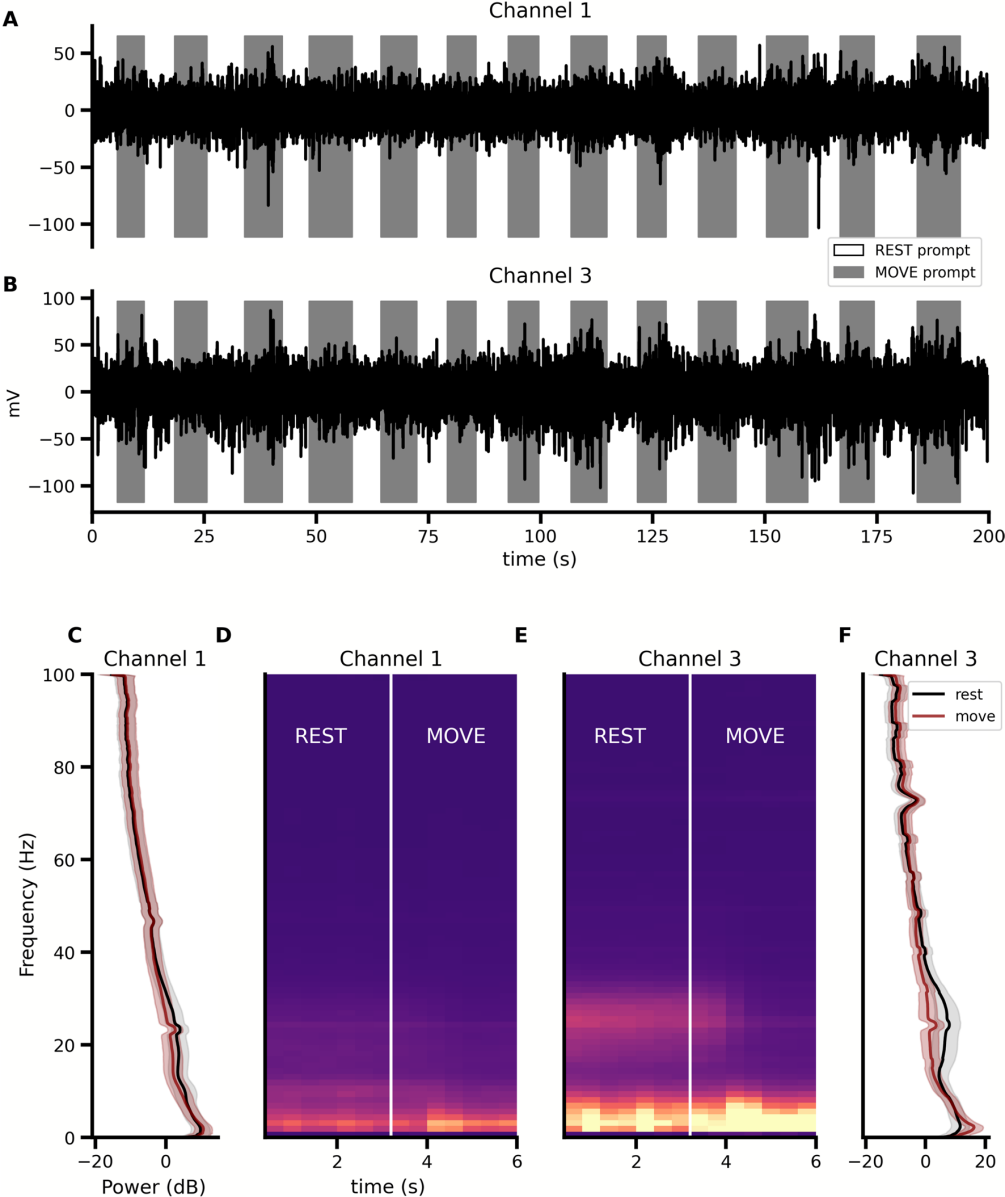
Remote ECoG Data collection. Results of data collected from the BCI system using the Activa PC + S Nexus device. **A**, **B** ECoG data from channels 1 and 3 and filtered through a 1 Hz high-pass filter. **C**, **F** Power spectra for channels 1 and 3 (shown as $\upmu \pm\upsigma$). **D**, **E** Time–frequency spectrograms of averaged windows (6.4 s, N = 2051) of data that show the changes in power of frequency bands between 1–100 Hz from filtered, averaged, and normalized data during multiple transitions from the REST state (indicated by the first half of the time series) to the MOVE state

The Activa PC + S provided four channels of data sampling. Channels 1 and 3 provided real-time data at 200 HZ, and channels 2 and 4 provided average power between 4–36 Hz sampled at 5 Hz. Figure 2a, b depicts 200 s of a 300 s representative trial after passing through a 1 Hz high-pass finite impulse response filter and labeled with the prompt presented at the time the signal was sampled. The power spectral density was then computed for each time channel for each packet to generate 321 spectral features for each time-series channel. The average power values between 4–36 Hz, collected from channels 2 and 4, were then grouped with these power values from the spectral estimates of the time channels to create a total of 644 features for each feature vector labelled with the prompt presented at the time of collection. The average spectral density demonstrates differences in the beta band (12–25 Hz) in each motor imagery state (Fig. 2c, f), and this is also observed over time (Fig. 2d, e). These feature vectors were used to train a classifier that first passed features through linear discriminant analysis, a 2-state hidden Markov model, followed by logistic regression to map the state probabilities to the motor imagery commands (see [49] for more detail).

Apart from data collection, daily use of the device was possible simply by turning the computer on and selecting the appropriate input and output devices using the App. Importantly, because we did not use any stimulation, safety concerns for at-home use were minimal.

### Graphical User Interface mobile application

**Fig. 3:**
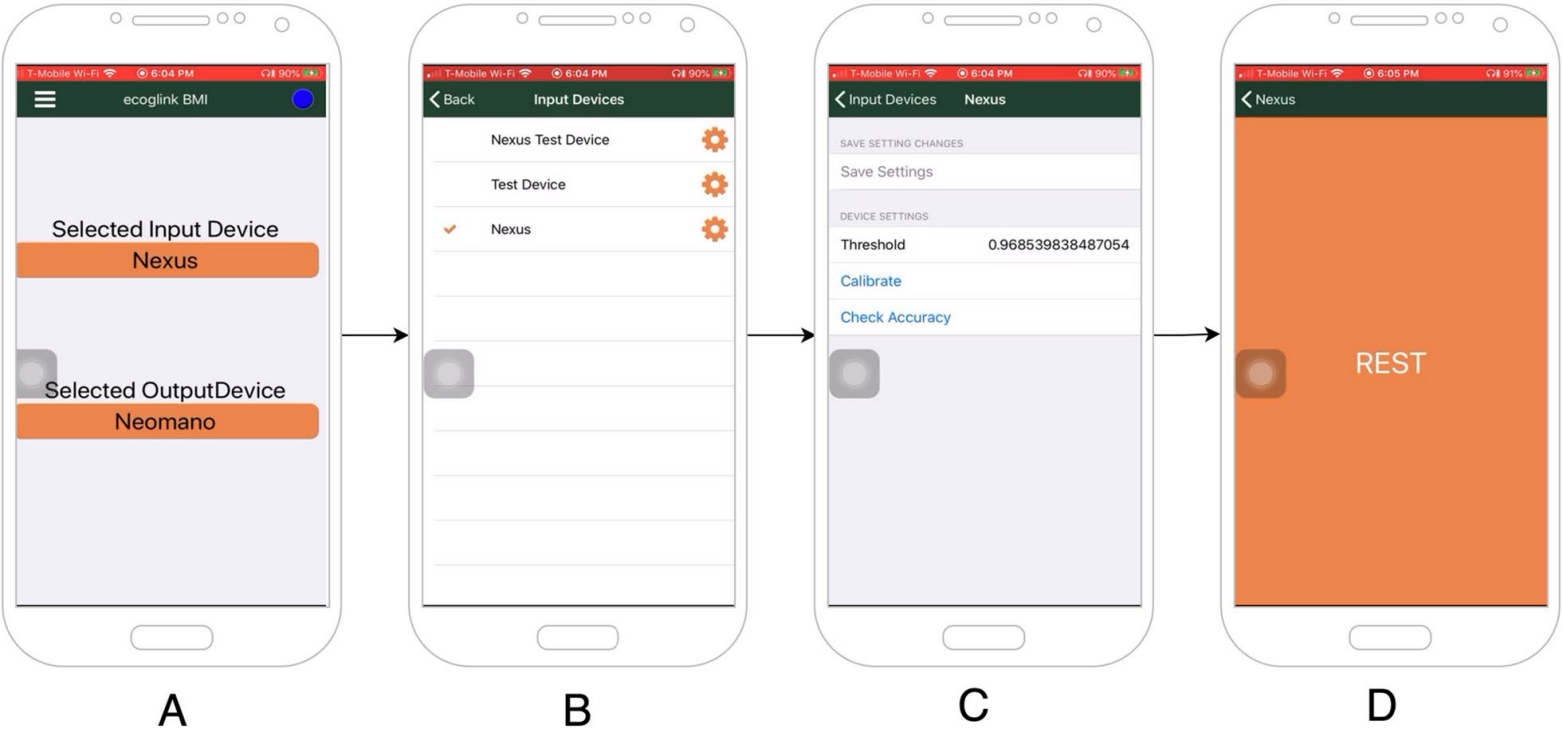
Mobile application overview. The App functioned as the GUI for the subject to interact with the BCI software running on the computer. **a** The home screen displayed the currently selected input and output devices in use. The blue dot in the upper-right marked the system’s status. **b** An input device selection screen, allowing the subject to select from more devices. **c** A settings page that allowed the subject to adjust parameters (such as decoder threshold, end effector motor speed, etc.) for a given device. These settings were defined by the software Class’s Application Programming Interface (API) on the computer’s end and were delivered over Bluetooth for dynamic display. **d** A data collection session that presented prompts to the subject either for assessing accuracy or applying calibration to the device’s decoder

We deployed a custom mobile phone application (App), developed with NativeScript, onto the subject’s smartphone allowing subject-control of the BCI system over BLE. The App provided a graphical user interface (GUI), displaying the current state of the BCI, allowing the subject to select available input and output devices, as well as alter the preferences and settings for each device (Fig. 3). The mobile phone application did not participate in any data acquisition or processing.

#### Dynamic display

The App displayed available input and output devices that could be selected for control (Fig. 3a, b). These devices would be added or removed from the App if they were connected or disconnected respectively from the minicomputer. When connected, devices provided device-specific settings that can be set using the App (Fig. 3c). Incorporating a new device into the system does not require an update to the App since it will dynamically display what the computer has access to.

#### Calibration

Neural signal motor imagery encoding can vary across subjects, therefore, machine learning algorithms must often be re-trained or re-calibrated to decode the subjects’ intended movement from the data. We designed the software application programming interface (API) to associate decoder training protocols and input devices. In this way, the subject could use the App to train a decoder for the Activa PC + S generated signal. When available, a calibration button is visible in the settings menu for that device (Fig. 3c). When selected, the App’s calibration mode is activated, allowing the subject to assess the accuracy of decoders associated with the given input device. While in calibration mode, the App receives text from the computer to display the patient to simultaneously record data and appropriately label the data to be used for testing or retraining the decoder via supervised learning models.

### Computer application

**Fig. 4:**
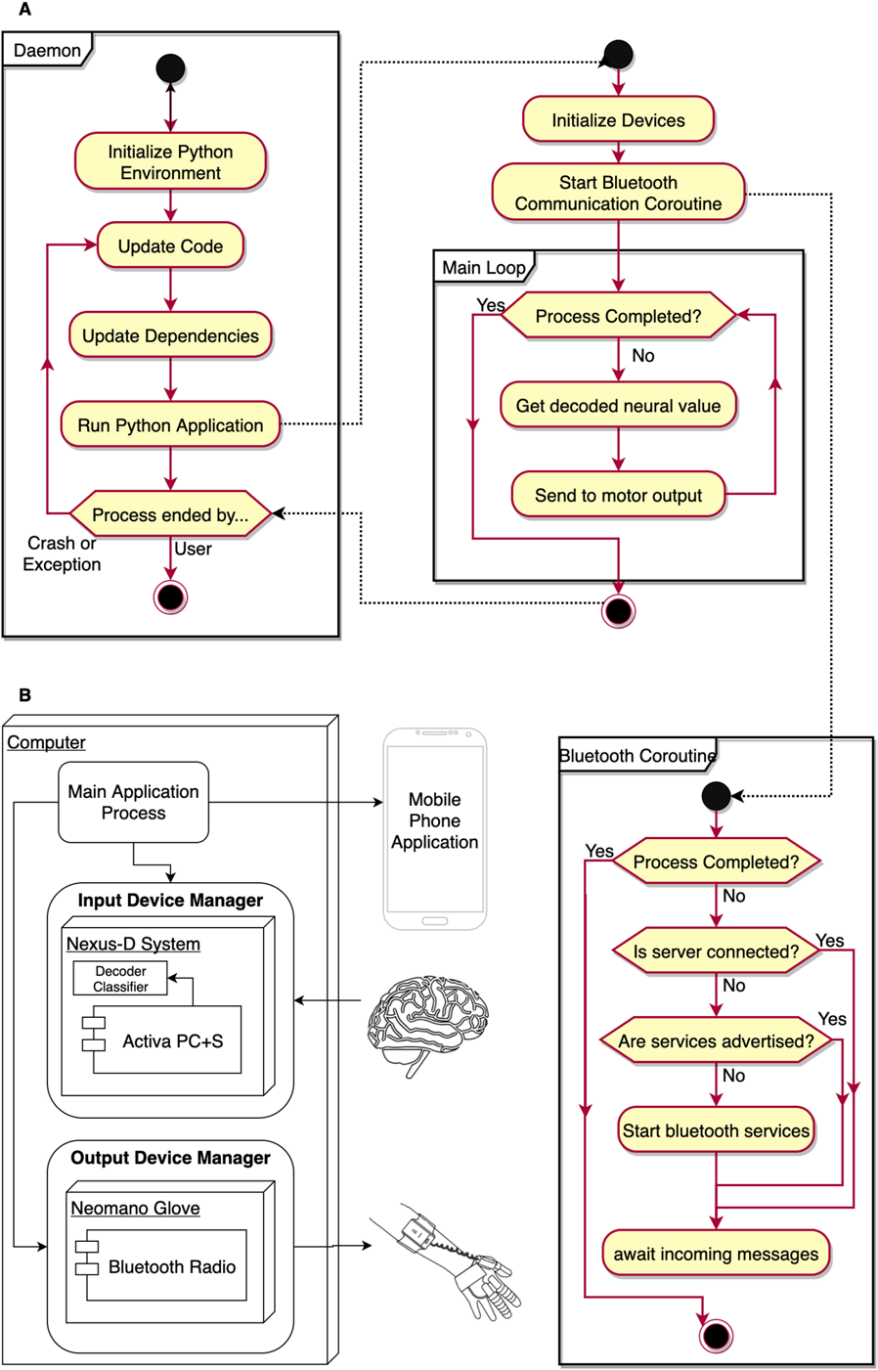
Application control flow. **a** The main application is initialized by a daemon script—or background running process—that ensured the program was always running while the computer was on. The computer application ran multiple coroutines asynchronously to allow for nearly uninterrupted data streaming between input and output devices as well as for Bluetooth communication. **b** The main application process iteratively made calls to classes that manage input and output devices. These device manager classes contained public methods for obtaining device input and sending commands to output devices. These device classes communicated with their hardware counterpart over serial port communication. Importantly, an array of devices may exist for the subject to use. These could be individually selected via the App over BLE

The computer application was written in Python [50–54] and consisted of two subprocesses: one for managing communication between input and output devices, the other for managing BLE communication with the App (Fig. 4a). These subprocesses used asynchronous event loops to control the points where execution could break and switch between these two processes to minimize processing delays that could occur between device communications. The main application process iteratively collected the decoded or classified neural signals from a selected input device and sent the returned command to the selected output device. Simultaneously, the Bluetooth process waited for incoming read, write, and notify requests from the App.

#### Modular design

A simple description of a motor BCI is an input device (or many of them) that provides neural data, transforms it into a meaningful output, and sends that output to an output device (or many of them). Because we assume that all devices in a BCI must communicate with the computer, our objective was to create an API to provide a framework for incorporating physically available hardware devices to be used by the BCI system. This was done by writing an abstract device class to generalize the methods and properties required for defining the base interaction and interface between a physical device and the BCI software (Fig. 4b). More specifically, by inheriting the methods and properties of the abstract device class, the methods for initializing the serial communication ports that enable the BCI system to communicate with the hardware are readily defined. With these device class files saved to a folder relative to the path of the application, the software was able to load these devices using the generic device class without needing to hard-code any device-specific details to the software directly. As an example, a Nexus class, coded for the Nexus-D telemeter, inherits this abstract device class, allowing the BCI system to automatically recognize the device and begin interacting with and monitoring data collected by the Activa PC + S. As mentioned briefly with the App, the API also provides the option to declare device attributes, if any, that the subject is allowed to adjust. By creating device setting objects, device classes could define how and to what extent each property could be changed. These device setting objects had inherited methods used to translate these settings into rendering commands that would be sent off and displayed by the App for end-user interaction. These device settings would allow the subject or caregiver to potentially change e.g., the speed, delay, or other user-centered preferences of a given device.

Together, this design provided the subject with dynamic hardware selection (e.g., mechanical orthosis, FES, etc.) and the ability to adjust device settings (e.g., speed or time delay of the orthosis, et.c) for more customized control. In our implementation, the Nexus telemeter used to capture data from the Activa PC + S, was encapsulated by inheriting the generic device class to create a programming interface to the hardware. The properties and methods of the signal decoder (Fig. 4b) associated with the Nexus telemeter, such as decoder threshold and decoder calibration, were supplied as device settings within the Nexus class. Because the decoder performs binary classification, a threshold was used to differentiate between the output movement probability that the decoded signal was that of MOVE or REST.

#### Bluetooth communication

We designed the software to communicate with the App using BLE, and created a cross-platform Bluetooth library to enable the computer to advertise services via Bluetooth peripheral role support (i.e., the Bluetooth host device). The data transmitted between the computer and the phone, including device settings, device status, and calibration, utilized three Bluetooth Generic Attribute (GATT) characteristics for data transmission. However, GATT characteristics have a minimum transfer unit of 512 bytes, and the size of the device settings information to transmit may exceed this limit. To circumvent this, we developed an additional layer over BLE to enable data streaming using a data queue to iteratively transferred data over a single BLE GATT characteristic. Bluetooth communication was only needed as the end-user made changes using the App. The Bluetooth library was designed using event-driven callbacks and asynchronous procedure calls. This design allowed the Python application to focus on collecting data from input devices, and delivering that data to output devices, only deferring to the Bluetooth communication process when called. By using Bluetooth, the BCI system could be used outside of the home, in the community since Bluetooth communication is independent of WiFi or internet connectivity.

#### Remote data collection

During use of the BCI, all data were recorded to files on the minicomputer hard drive. The software saved the data files to a directory that automatically synchronized the university’s encrypted HIPAA compliant cloud storage, allowing almost instantaneous access to the incoming data, assuming the on-board computer had an active internet connection. To ensure that the computer would have an internet connection, we saved the subject’s home WiFi’s service set identifier (SSID), security profile, and password onto the computer before installing the device onto the subject’s wheelchair. This configuration allowed the computer to connect to the subject’s home WiFi to allow for data synchronization once the subject arrived home.

### Deployable for at-home use

The goal for the system design was to provide a subject, who was equipped with a fully implanted neurosensory device, with a functional BCI for at-home use. We deployed the system to the home by installing the designed software on a minicomputer (m90n Nano, Lenovo, China; Windows 10 Pro; Intel i3 2.10 GHz, 8 GB RAM) and housed the computer, along with a lithium battery (50,000 mAh power bank, Krisdonia, China) in a custom 3D-printed case. This case was attached to the back of the subject’s wheelchair by using straps that looped through holes printed onto the exterior of the case. Sliding doors were placed on two sides of the case for easy access to charging ports and power buttons on the battery and minicomputer. After installing the BCI software onto the minicomputer, we configured the computer to run the software at startup by invoking a daemon process that would ensure the software would always be running (Fig. 4a). We stored the code for our software online using a version control system. This allowed our daemon script to pull from the version control system to ensure the code was up to date. This setup allowed us to use git commit dates and timestamps as conditions against which we could programmatically select and analyze data collected under varying software conditions (e.g., before and after decoder updates or initial bug fixes).

## Results

### Bench testing

#### Bluetooth communication delays

**Fig. 5:**
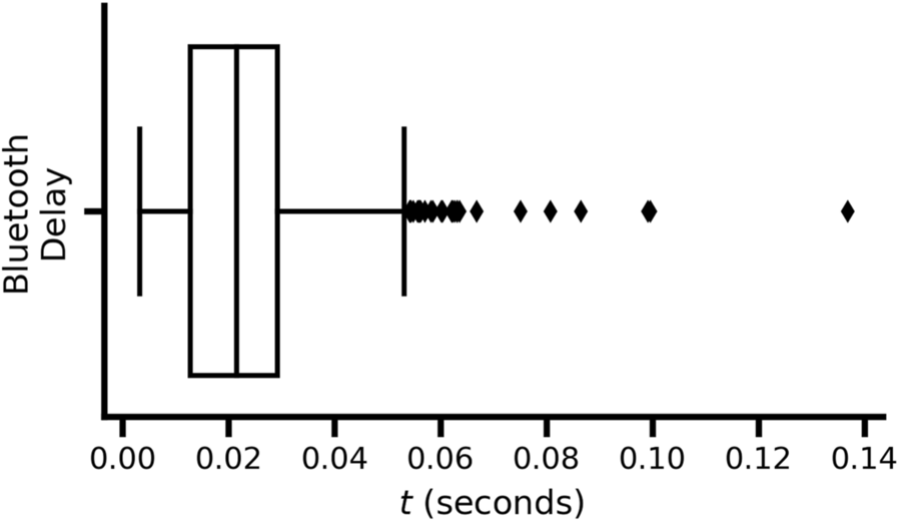
Bluetooth low energy communication Benchmark. The time delay observed during a data collection session (n = 750). Bluetooth transmission time delay was measured as the difference between the time at which the display prompted changed on the App and the time the prompt was changed on the computer system to initiate BLE (prompt to display notifying characteristic)

The Activa PC + S has a sampling rate of 200 Hz and has an onboard memory store that is overwritten at a rate of 2.5 Hz (400 ms). API calls to the Activa PC + S, via the device’s firmware, block program execution until data is available. Importantly, if the time between two subsequent calls to the API to collect data is > 400 ms, the data sampled by the intracortical electrodes during the time between the two API calls will be lost, since the Activa PC + S will already be storing data for the next 400 ms block by the time the second call is issued. Thus, significant latencies between calls to collect data could result in data loss. This is particularly important for data collection for decoder training, where incoming data must be synchronously labeled. Because data labeling relied on messages sent over BLE, we needed to ensure the time to display the movement instruction to the subject temporally matched up with the data being collected. Thus, data can only reliably match up with the onscreen instruction labels presented to the subject if the time to send the instruction to the App over BLE was less than 400 ms to minimize the chances of overwriting data as well as subject reaction times. By recording time stamps during a simulated recalibration session, the difference between the time stamp at which the prompt was sent by the computer, and the time stamp at which the same prompt was displayed on the App, demonstrates that the average delay-to-screen was 23 ms (Fig. 5), indicating that the Bluetooth communication was not adding time delays that would affect data collection. We also found that no Bluetooth data packets were dropped during transmission.

#### Software profiling

**Fig. 6:**
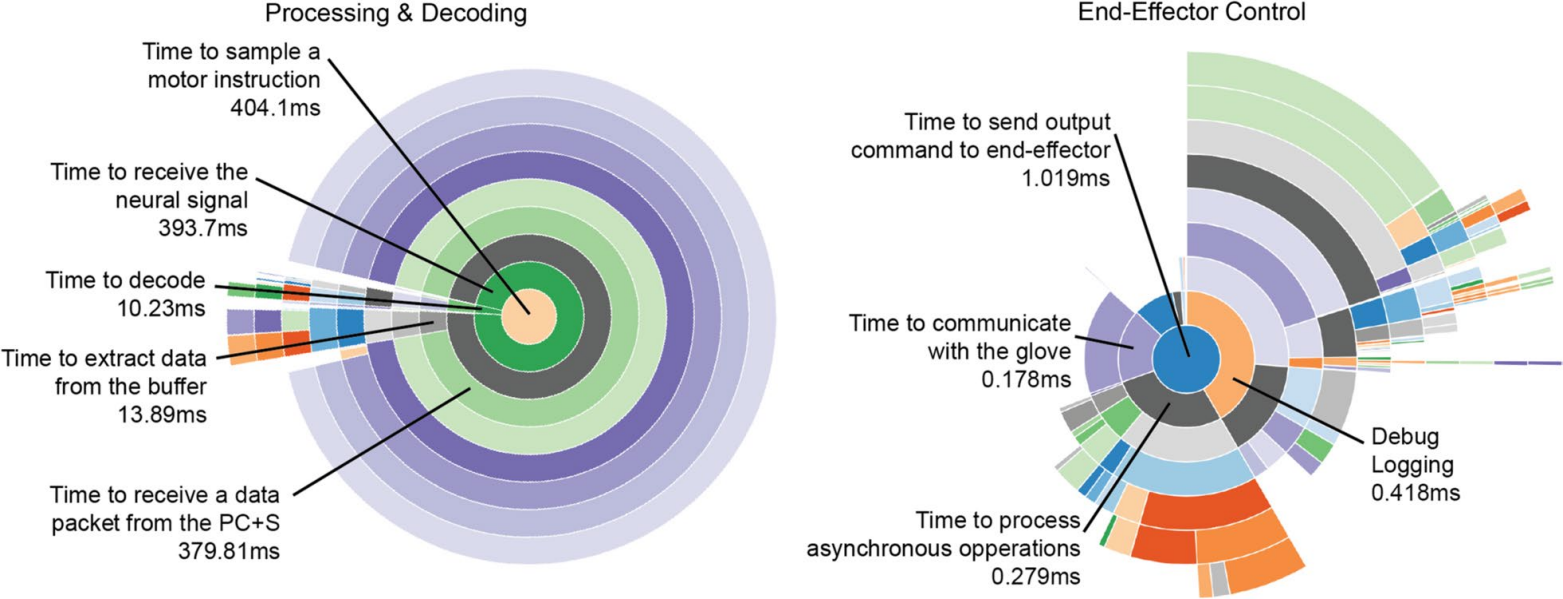
System profiling. Sunburst diagrams representing the proportions of time spent to process incoming data (left) and send the decoded output to the glove (right). The center of each sunburst diagram represents the process to obtain the decoded neural signal (left), or to send the command to the glove (right). Each arc surrounding the center point represents a subprocess needed to be carried out to process incoming data (left) or send data (right). The length of the arc represents the proportion of time taken for a subprocess to complete relative to subprocesses that depend on it for completion. While there are many subprocesses, those relevant to the BCI software are highlighted. The remaining subprocesses are system sepectific processes such as input–output operations

System application profiling was measured using the cProfile Python module and visualized using SnakeViz (Fig. 6) to characterize the latency caused by processes in the BCI system. The BCI software was initialized after the subject was set up at home. Profiling was collected while the system was in use for 5 min. The main application loop consisted of two main functions: (1) reading in decoded commands from the selected input device (Fig. 6 left) and (2) sending the commands to the selected output device (Fig. 6 right). System profiling revealed that the time to process incoming signals into a motor instruction command was approximately 400 ms (Fig. 6 left), after which only 1 ms was spent sending that command to the end effector for hand grasp (Fig. 6 right). The first portion of this main loop: processing the incoming signal into a motor instruction, was made up of two subprocesses: (1) receiving the neural channel data from the Activa PC + S (~ 393 ms), and (2) decoding that signal into a motor command (10.23 ms). Then, sending that command off to the Neomano glove required approximately 1 ms.

### Home testing

#### Signal decoding and classification

**Fig. 7:**
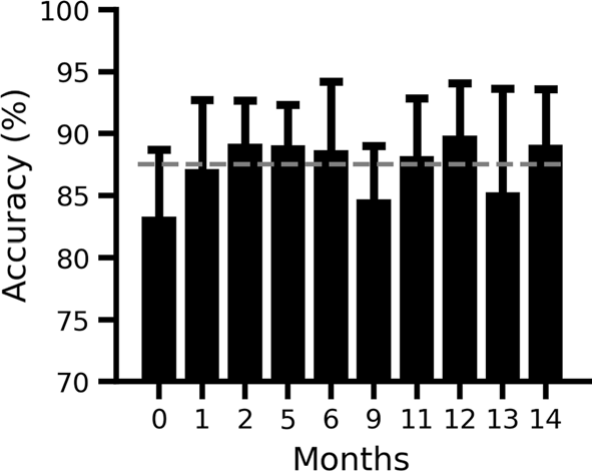
Decoder classification. Classification performance of the decoder associated with the Nexus telemeter input device. Month 0 indicates assessment of training data and the subsequent months indicate the number of months since training. The black dotted line indicates the global median of 87.53%

Accuracy metrics for the motor imagery classifier continued to be measured over the next year after training without recalibration (Fig. 7). Decoder accuracy defined as the number of correctly classified windows of data, remained stable with a median accuracy of 87.53% across 79 trials (Fig. 7; gray dotted line).

### Caregiver management

**Fig. 8:**
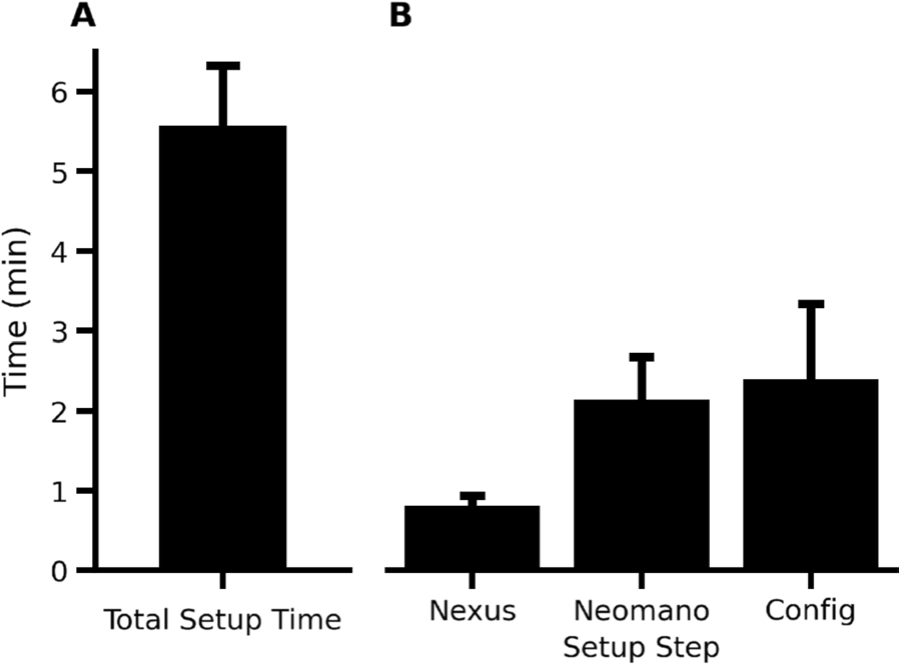
System setup time. Elapsed time taken by the subject’s primary caregiver to set up the system. Repeated measurements once a day for several days. **A** Presents the total time to setup the system, while **B** presents the times for the different set up steps that sum to the total elapsed time. Calibration time is not included because it was not necessary during datily setups

Little to no training for caregivers was required to learn to setup the device for the subject using our BCI implementation. Set up merely required turning the system on, appropriately positioning the telemetry antenna on the subject, and fitting any end-effector devices onto the subject’s limb. From this point, the subject was able to configure, calibrate, and control the BCI system using the App without assistance. To quantify this, we measured the average amount of time elapsed between the time the nurse began setting up the system up to the moment the subject had neural control of the mechanical glove (Fig. 8a). On average, time to setup this implementation was 5.58 min, with most of this time (2.34 min) taken to establish a Bluetooth connection between the App and the minicomputer and configure the system as well as to don the Neomano glove (2.18 min).

## Discussion

Our design aimed to allow the BCI to function both in and out of the home and give the subject control over device selection, data collection, and system settings and preferences, all while minimizing the need for caregiver assisted setup. Although this system was implemented in a subject with SCI with an implanted neural interface, applications can be extended to other forms of paralysis, or wherever BCI can be employed, for example in use with EEG headsets or intracortical spike signals.

### Mobile phone application

In our design, the App functioned as a GUI. However, other implementations have used smartphones for signal processing—using the phone as the sole processing unit for the system [55–58]—as opposed to using an on-board minicomputer. Others have used smartphones to drive communication with home appliances [35, 36]. Here, the use of NativeScript limited the development of the phone application to Bluetooth Low Energy protocols, which limits our system’s ability for high bandwidth data streaming that Bluetooth classic radio frequency communication (RFCOMM) could otherwise achieve for raw signal transmission. The alternative solution of using classic RFCOMM, however, would likely drain the battery of the phone fairly quickly, as reported in Campbell et al. [56]. However, the implementation of RFCOMM would need to be considered for continuous and reliable data collection and streaming if the system were to be moved entirely onto the phone in future iterations. Additionally, migrating to phone-use warrants increased security measures. As [59] points out, widespread and trusted use of such systems will require care to ensure the integrity and security of the system.

As an important note, our subject was able to navigate use of the App using the residual movements available with their bicep muscles. However, the App is simple enough to enable a caregiver to configure or alter system settings where needed, and doesn’t limit the application of our system to subjects with unique residual movements.

### Portable system

For more practical use of BCIs outside of the laboratory, system portability is a significant component [27]. One method for increasing portability, is using smaller computational equipment that has become more readily available in personal tablets, phones, and minicomputers. Several previous studies have used portable devices such as tablets and phones for command [35] and signal processing [55, 57, 58], subject interaction [38], and as the end effector itself [56]. For our design, we used a minicomputer and the subject’s smartphone, which allowed us to give the subject control of the BCI while ensuring that the BCI software could continue running independently of the phone. Additionally, by way of cost, these components are already consumer grade products that can readily be purchased. The short-range Bluetooth communication used by the phone and motorized orthotic allowed for constant control of the system, which permitted the subject to continue using the BCI outside of the home. Because the motorized orthotic communicated with the system over wireless Bluetooth radio, WiFi was only necessary for uploading data needed for offline analysis, the use of the BCI system itself ran independently of internet connectivity.

### Modular system

Modular BCI designs for research development have helped improve scientific reproducibility and have reduced the time required to develop new BCI software and configurations. Several software tools, packages, and pipelines have been developed to help with this effort [60–64]. Such applications have been used successfully in the lab and at home using some of these systems [65]. In addition to these tools, other studies have shown a variety of successful output controls including, cursor control, spellers, home appliance control, exoskeletons, prosthetics, and FES [6–8, 11–24, 47]. Our design focused on end-user interaction while enabling the software to scale across OS and processor architectures. While we only utilized a motorized orthotic when testing our device, we ensured the system could be modular, including dynamic changes to input and output devices at runtime. This modularity was another purpose for employing the subject’s mobile device. It allowed the subject to swap end effector devices, initiate training sessions, and adjust available system settings.

Input devices (e.g., EEG system) have plug-n-play functionality, when a device class is provided. Implementing a device class requires some mechanism for sampling digitalized data from the device to the computer; e.g., through serial port, socket, or device-provided API. A decoder from our work can be used or custom made that transforms, classifies, or regresses the input signal into a meaningful value. The device class must inherit the device base class and define a get_input method that then (1) collects the data as previously determines, (2) decodes that data into a meaningful value, then (3) returns that value. Lastly, serial port, socket, or API properties can be defined on the class that the platform will utilize to automatically detect the device during start up. With these in place, a new device can be added to the platform, and will be available for selection on the mobile app for use.

### Subject interaction with the at-home BCI platform

Together, the BCI platform and its implementation not only enabled a subject with cervical SCI to reanimate hand grasp at home and swap modular components, but it also gave the subject control over the settings and preferences for each device individually and the BCI system as a whole. More specifically, these settings included options for changing the response time of the motorized orthotic and restarting the BCI application. Researcher assistance or transport to the laboratory became unnecessary because the App allowed the subject to initiate data collection sessions on their schedule.

The deployment of our system for use at home minimized the need for complex donning and doffing procedures. Over several sessions, we measured the average setup time, from start until the subject could control the glove, to be around 5 min. This is well within range of previously recommended setup times of 10–20 min desired by surveyed potential users [48]. A large part of this simplification is likely a result of using a fully implanted device. This setup avoids the use of EEG caps that might require wire connections, gel application, accurate placement, and configuration of the EEG cap [66, 67]. In comparison, a recent study was able to achieve an average setup time of 20 min in 8 caretaker-subject pairs over several sessions [68]. In general, setup times are dependent on the setup time of each component of a BCI system. Continued developments in non-invasive technologies will help drive down these setup times and configuration complexities. Although, user-evaluation was not systematically evaluated the subject did comment: “It’s easy to use and control and only sometimes take a bit of time to connect over Bluetooth”.

Though our implementation only utilizes 4 ECoG channels, potentially limiting the functional output of the system in comparison to many EEG channels, the purpose of this implementation was to minimize the setup time and complexity to use at home, while still delivering some functionality to the subject. Still, even fully-implanted systems can require a wired setup that requires plugging in the system [6–8, 19, 47].

In our implementation, however, switching on the battery fixed to the back of the wheelchair was sufficient to start up the system. From here, aligning the telemetry antenna to the subcutaneous transmitter enabled neural control. Notably, the ease of setup because of using an implanted device such as the Activa PC + S is a matter of implementation of this BCI platform rather than a requirement to use the platform. The platform could be implemented using an EEG headset. For the implementation presented here, the fully implanted Activa PC + S was used to improve at-home setup and use of the system as a whole.

### Limitations

Python as the development language may not result in a completely OS agnostic platform. This limitation is mostly a result of methods the language uses to interface with the OS and hardware. An alternative solution could be to utilize already available software such as BCI2000 [60]. However, using an interpreted language such as Python for development removed the need for compile time during remote system updates.

The subject in the implementation presented here was able to use the App GUI on their own using their remnant bicep function. This generally limits the GUI’s use for less abled subjects. However, the App GUI still provides convenient system access for the caregiver. Additionally, future iterations are planned to enable voice activation and/or gaze control to enable better control in subjects with varying disability and control of the hand and arm.

Using the Activa PC + S and Nexus telemeter isn’t completely wireless. Data sample collection between the Nexus telemeter and the Activa PC + S requires an external antenna to be placed close to the implanted generator. However, Newer implementations of implantable generators (Medtronic Percept, St. Jude, Boston Scientific, Clinatec, etc.) are building wireless capabilities into their generators. Additionally, the Activa PC + S, used in cases of deep brain stimulation, can last up to 5 years, but the battery life of the device used in this context where stimulation is not used, is unknown. Implants that can be inductively charged [69] and wirelessly transmit their signals [70] will provide a more robust system that further minimizes system maintenance and caregiver assistance while maximizing portability.

Like many invasive studies, our implementation of an invasive device is limited to one subject, and thus difficult to generalize. Fortunately, using event-related desynchronizations as a method to perform binary classification of motor imagery is well established [68, 71–74], and a few studies have employed it in the home [34, 68, 75, 76]. Although, we employed this BCI system for use in a subject with cervical SCI, it has provided insights in to limitations how the software and App can be improved for easier incorporation into other BCI implementations.

## Conclusion

Future adoption of BCI-related technology will need to ensure that BCI systems are portable, intuitive to setup, and simple to configure [48]. This modular BCI software design puts forth a light-weight platform for implementing BCI systems on consumer computer platforms and mobile phone devices. The BCI platform enables easy addition of input and output devices that the subjects can easily switch between using the mobile graphical user interface. The implementation of the BCI modular software platform design demonstrates the increasing feasibility of transitioning BCI systems to more portable units for at-home use. As more assistive and rehabilitative device become available, modular platforms may provide more functionality for BCI users. The development of such systems will make the assistive and rehabilitative capabilities of BCI more accessible to subjects who would benefit from them. BCI systems in the home settings provide fertile opportunity to improved the independence in subjects with paralysis. Future work will focus on expanding customized mapping between input and output devices to allow for use of multiple devices simultaneously.

## Data Availability

Individual participant data that underlies the results reported in this article after de-identification, study protocol, statistical analysis plan, and analytic code will be made available upon request to researches who provide a methodologically sound proposal.

## Abbreviations

BCI: Brain–computer interface
SCI: Spinal-cord injury
RF: Radio frequency
EEG: Electroencephalography
ECoG: Electrocorticography
BLE: Bluetooth low energy
RFCOMM: Radio frequency communication
App: Mobile phone application
GUI: Graphical user interface
OS: Operating system
